# Prospective intraindividual comparison of a standard 2D TSE MRI protocol for ankle imaging and a deep learning-based 2D TSE MRI protocol with a scan time reduction of 48%

**DOI:** 10.1007/s11547-023-01604-x

**Published:** 2023-02-19

**Authors:** Gabriel Keller, Arne Estler, Judith Herrmann, Saif Afat, Ahmed E. Othman, Dominik Nickel, Gregor Koerzdoerfer, Fabian Springer

**Affiliations:** 1 Department of Diagnostic and Interventional Radiology, University Hospital Tübingen, Eberhard Karls University Tübingen, Hoppe-Seyler-Str. 3, 72076 Tübingen, Germany; 2 Universitätsklink für Neuroradiologie, Johannes Gutenberg-Universität Mainz, Mainz, Germany; 3 MR Applications Predevelopment, Siemens Healthcare GmbH, Allee Am Roethelheimpark 2, 91052 Erlangen, Germany; 4 Department of Diagnostic Radiology, BG Trauma Center Tübingen, Eberhard Karls University Tübingen, Schnarrenbergstr. 95, 72076 Tübingen, Germany

**Keywords:** Deep learning, Artificial intelligence, MRI, Ankle, Machine learning

## Abstract

**Purpose:**

Magnetic resonance imaging (MRI) scan time remains a limited and valuable resource. This study evaluates the diagnostic performance of a deep learning (DL)-based accelerated TSE study protocol compared to a standard TSE study protocol in ankle MRI.

**Material and methods:**

Between October 2020 and July 2021 forty-seven patients were enrolled in this study for an intraindividual comparison of a standard TSE study protocol and a DL TSE study protocol either on a 1.5 T or a 3 T scanner. Two radiologists evaluated the examinations regarding structural pathologies and image quality categories (5-point-Likert-scale; 1 = “non diagnostic”, 5 = “excellent”).

**Results:**

Both readers showed almost perfect/perfect agreement of DL TSE with standard TSE in all analyzed structural pathologies (0.81–1.00) with a median “good” or “excellent” rating (4–5/5) in all image quality categories in both 1.5 T and 3 T MRI. The reduction of total acquisition time of DL TSE compared to standard TSE was 49% in 1.5 T and 48% in 3 T MRI to a total acquisition time of 5 min 41 s and 5 min 46 s.

**Conclusion:**

In ankle MRI the new DL-based accelerated TSE study protocol delivers high agreement with standard TSE and high image quality, while reducing the acquisition time by 48%.

## Introduction

The ankle is the second most injured body part in sports [1, 2] and commonly evaluated by magnetic resonance imaging (MRI). Ongoing issue thereby is the acceleration of acquisition time. For musculoskeletal imaging, usually multiple two-dimensional (2D) multi-slice acquisitions of anisotropic voxels [3] are used. Previous studies tested three-dimensional (3D) sequences with isotropic voxels in ankle imaging with secondarily reconstructed slice orientations as a method for acceleration [4–9]. However, the positive correlation of image quality and acquisition time leads to an acquisition time of about 10 min for a 3D data set with small voxel sizes [10]. Furthermore, artifacts may corrupt the whole data set which might be seen only post-acquisition and require a repeated examination.

An established approach for accelerating the image acquisition is to acquire undersampled data and perform a parallel imaging (PI) reconstruction [4, 5, 11]. The approach is hereby limited by a decreased signal-to noise ratio (SNR) and an increased level of artifacts [12]. A further approach is compressed sensing (CS) [13–15] which combines an incoherent undersampling scheme with a sparsity enforcing reconstruction to recover the undersampled data. Limitations are residual blurring and an unrealistic image presentation.

Deep learning (DL) algorithms for image reconstruction from undersampled MRI data deliver a method to overcome these problems. They offer the potential of image reconstructions with higher SNR compared to conventional reconstruction techniques while at the same time delivering a realistic image presentation [16]. The gain in SNR can be spent on a reduction of the acquisition time, improved image quality or combinations thereof [17]. Recent studies have shown promising results in the knee [12].

The purpose of this study was to evaluate the diagnostic performance of DL-based PD- and T1-weighted turbo spin echo (TSE) sequences in a routine study protocol for ankle imaging at 1.5 T and 3 T MRI in a prospective setting.

## Materials and methods

This study was approved by the institutional review board (Eberhard Karls University Tuebingen, project identification code: 055/2017BO2). The study was conducted in accordance with the Declaration of Helsinki (as revised in 2013).

### Study population

The study period of this prospective single center study was October 2020 till July 2021. All patients who underwent clinically indicated MRI of the ankle were included. Excluded were patients with divergent study protocols due to the specific clinical problems, e.g., follow-up care of tumor patients.

### Hardware parameters of the MRI acquisition

The examinations were performed in random selection either on a 1.5 T scanner (MAGNETOM Aera or MAGNETOM Avanto^fit^, Siemens Healthcare, Erlangen, Germany) or on a 3 T scanner (MAGNETOM Skyra, MAGNETOM Prisma^fit^ or MAGNETOM Vida, Siemens Healthcare, Erlangen, Germany). All patients were examined in supine position, feet first using a dedicated 16-channel foot-ankle coil (Foot/Ankle 16, Siemens Healthineers, Erlangen, Germany).

### Standard MRI protocol for ankle imaging

**Fig. 1:**
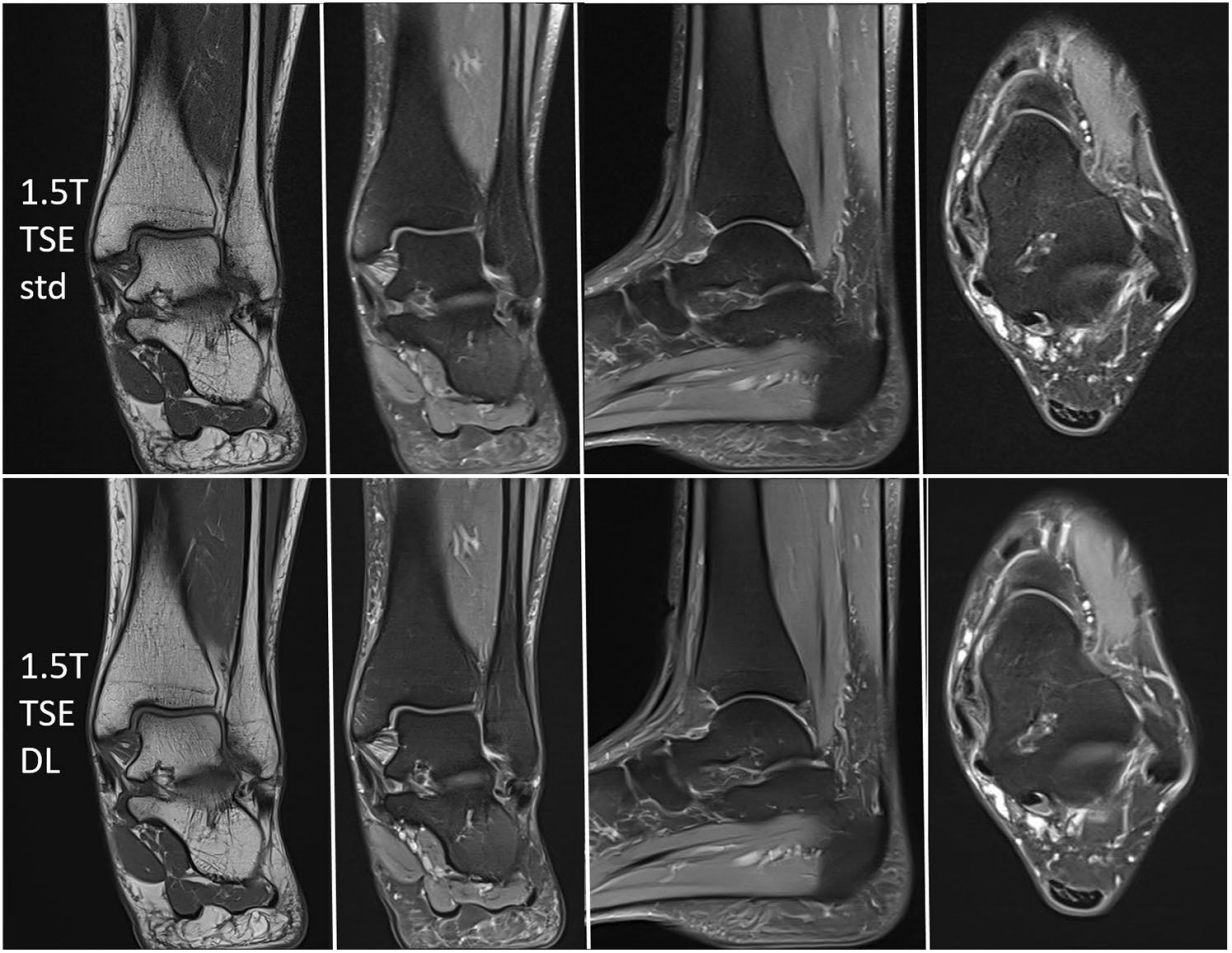
Exemplary MRI (left ankle, 45 years, female) examined with a standard 2D TSE protocol (upper row) and the new DL-based 2D TSE protocol (lower row) on a 1.5 T scanner (from left to right: T1 coronal, PD with fat saturation coronal, sagittal, transversal). T = Tesla, TSE = turbo spin echo, DL = deep learning, std = standard

**Fig. 2:**
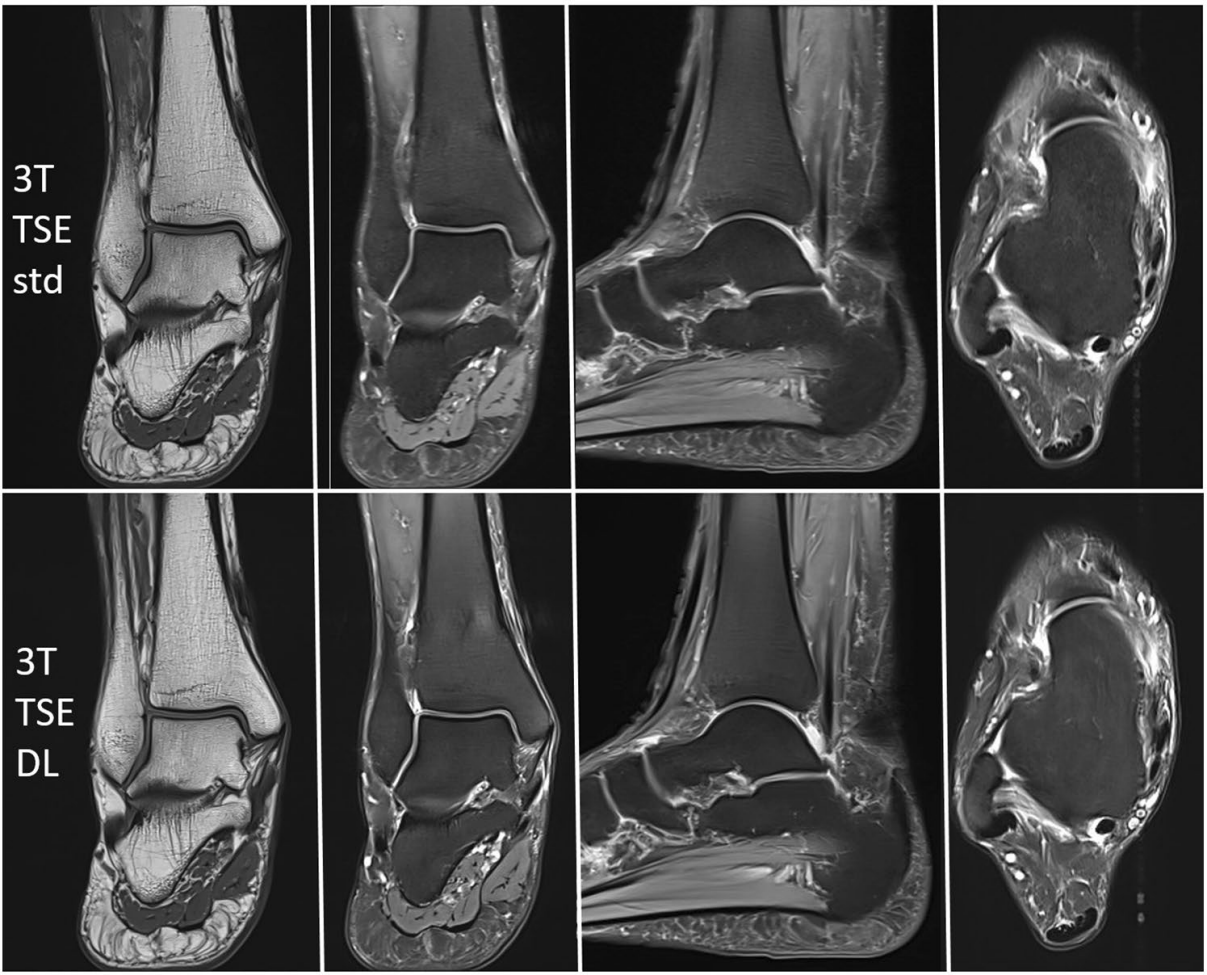
Exemplary MRI (right ankle, 63 years, female) examined with a standard 2D TSE protocol (upper row) and the new DL-based 2D TSE protocol (lower row) on a 3 T scanner (from left to right: T1 coronal, PD with fat saturation coronal, sagittal, transversal). T = Tesla, TSE = turbo spin echo, DL = deep learning, std = standard

According to international guidelines (e.g., European Society of Musculoskeletal Radiology) our study protocol consisted of a coronal T1-weighted TSE sequence and coronal, sagittal and transversal PD-weighted TSE sequences with spectral fat suppression (see Figs. 1, 2). Total acquisition time of the standard study protocol was 10 min 59 s on a 1.5 T scanner and 11 min 0 s on a 3 T scanner. Further imaging parameters are displayed in Table 1.

**Table 1:** Acquisition parameters of standard TSE and DL-based TSE ankle MRI

Field strength (T)	Sequence	Fat saturation	Orientation	TA (min:sec)	Voxel size, acquired (mm^3^)	Slices	FOV (mm)	TE (ms)	TR (ms)	FA (°)	Av	*C*	PAT	TF
1.5	PD TSE std	FS	Coronal	2:25	0.60 × 0.42 × 3.0	28	160	30	3090	150	2	1	3	5
	PD TSE DL			1:09	0.58 × 0.42 × 3.0	28	160	41	3000	150	1	1	3	5
	PD TSE std	FS	Transversal	2:44	0.56 × 0.39 × 3.0	36	150	33	4000	150	1	1	2	5
	PD TSE DL			1:45	0.55 × 0.39 × 3.0	36	150	44	3640	150	1	1	3	5
	PD TSE std	FS	Sagittal	3:15	0.60 × 0.42 × 3.0	22	160	30	3000	150	2	1	3	5
	PD TSE DL			1:45	0.58 × 0.42 × 3.0	22	160	27	3000	150	1	1	3	5
	T1 TSE std	None	Coronal	2:35	0.39 × 0.31 × 3.0	28	160	11	457	150	2	3	3	3
	T1 TSE DL			1:02	0.43 × 0.31 × 3.0	28	160	11	552	150	1	3	2	3
3	PD TSE std	FS	Coronal	2:25	0.36 × 0.36 × 3.0	28	160	32	3090	150	2	2	3	5
	PD TSE DL			1:03	0.36 × 0.36 × 3.0	28	160	43	3000	150	1	2	6	5
	PD TSE std	FS	Transversal	2:52	0.36 × 0.36 × 3.0	36	150	34	4200	150	1	2	2	5
	PD TSE DL			1:46	0.38 × 0.36 × 3.0	36	150	47	3800	150	1	2	4	5
	PD TSE std	FS	Sagittal	3:02	0.31 × 0.31 × 3.0	24	160	32	3090	150	2	1	2	5
	PD TSE DL			1:26	0.30 × 0.31 × 3.0	24	160	29	3100	150	1	1	4	5
	T1 TSE std	None	Coronal	2:41	0.31 × 0.31 × 3.0	28	160	13	476	150	2	4	3	3
	T1 TSE DL			1:31	0.28 × 0.31 × 3.0	28	160	13	476	150	1	5	6	3

*TSE* turbo spin echo, *DL* deep learning, *TA* acquisition time, *FOV* field of view, *TE* time to echo, *TR* time to repeat, *FA* flip angle, *Av* averages, *C* concatenations, *PAT* parallel acquisition technique factor, *TF* turbo factor, T Tesla, *PD* proton density, *std* standard

### DL-based MRI protocol for ankle imaging

As in PI a conventional equidistant undersampling pattern was used as it provides equal performance with DL-based reconstruction in contrast to incoherent sampling patterns known from CS. The basis for the prototypical reconstruction is a fixed iterative reconstruction scheme comprising a variational network [18–20]. Inputs to this DL-based reconstruction are k-space data, bias field correction and coil sensitivity maps. Multiple cascades made up from a data consistency step using a trainable Nesterov momentum and a following convolutional neural network regularization constitute the DL-based reconstruction. The parameters of the reconstruction where obtained through supervised training using volunteer data acquired on different 1.5 T and 3 T scanners (MAGNETOM, Siemens Healthcare, Erlangen, Germany). The reconstruction method is described in more detail in [16].

Total acquisition time of the DL-based study protocol was 5 min 41 s on a 1.5 T-scanner and 5 min 46 s on a 3 T-scanner (see Figs. 1, 2). Further imaging parameters are displayed in Table 1.

### Evaluation of the image quality

The image quality was evaluated independently by two radiologists (six years of experience on MSK imaging including fellowship training; five years of experience on MSK imaging) on a 5-point Likert scale (1 = “non-diagnostic image quality”, 2 = “low image quality”, 3 = “moderate image quality”, 4 = “good image quality”, 5 = “excellent image quality”). Criteria were image noise, sharpness, banding artifacts and diagnostic confidence.

### Evaluation of the diagnostic accuracy

The readers rated the MRI blinded to patients’ data, acquisition technique, magnetic field strength of the scanner and in random order. The relevant ligamentous structures were graded into four groups from intact to complete rupture, posttraumatic fiber alterations such as thickening were classified as 1. The relevant tendinous structures were also graded into four groups from intact to complete rupture with inflammatory or reactive changes such as pathological surrounding fluid or hyperintense signal of the tendon on PD-images were classified as 1. Cartilage defects and osteochondral lesions were graded as per International Cartilage Regeneration and Joint Preservation Society (ICRS) and Kramer’s classification [21]. Other pathological findings were rated dichotomously. In addition, a consensus reading of the standard TSE examinations was carried out to determine the presence of pathologies in the study collective.

### Statistical analysis

Statistical analysis was performed using the software packages JMP (Version 15.2.0, SAS Institute, Cary, NC, USA) and SPSS (Version 28.0.0.0, IBM Corp., Armonk, NY, USA). Ordinal variables such as image quality criteria are reported as median with range, correlations were analyzed by likelihood ratio. *p* values < 0.05 indicate statistical significance. Cohen’s Kappa was used for the agreement between standard TSE and DL TSE MRI, values of 0.00–0.20 were considered as slight, 0.21–0.40 as fair, 0.41–0.60 as moderate, 0.61–0.80 as substantial, and 0.81–1.00 as almost perfect/perfect levels of agreement [22].

## Results

### Study population

A total of 51 consecutive patients underwent ankle MRI in the study period October 2020 till July 2021. Four patients (7.8%) were excluded due to deviant study protocols. The final sample size was *n* = 47 (26 female/21 male). 25 examinations were performed on a 3Tscanner and 22 examinations on a 1.5 T scanner. Mean age of the final study population was 38.02 [± 14.96] years.

**Fig. 3:**
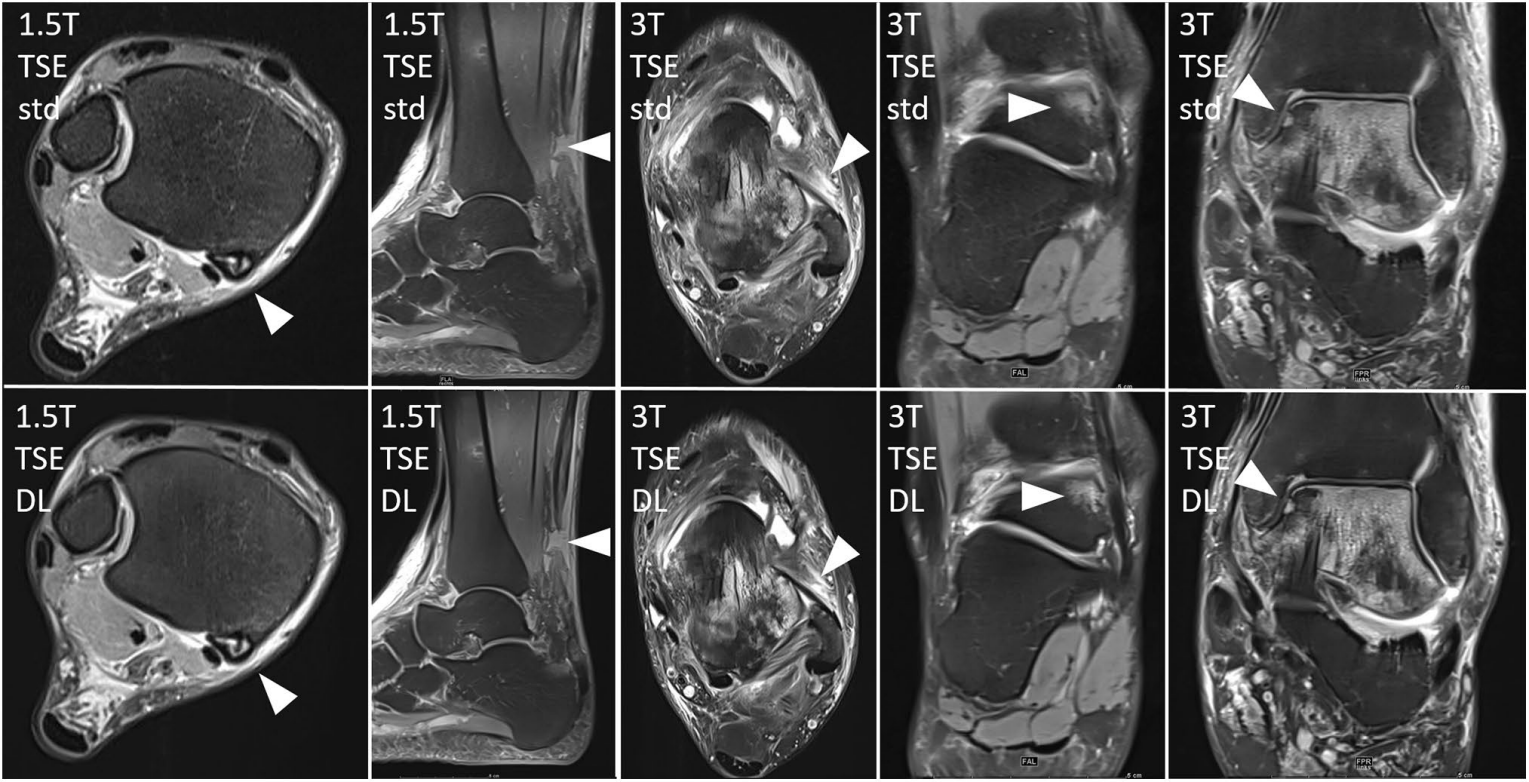
Exemplary lineup of structural pathologies in standard TSE (upper row) and DL-based TSE (lower row) 1.5 T and 3 T MRI. From left to right: tendinitis of the tibialis posterior muscle (right ankle, 47 years, female), complete intratendinous rupture of the Achilles tendon (right ankle, 46 years, male), intact ATFL with surrounding edema (left ankle, 40 years, male), bone marrow edema of the talus (right ankle, 46 years, female), cartilage lesion of the talus (left ankle, 40 years, male). T = Tesla, TSE = turbo spin echo, DL = deep learning, std = standard, ATFL = anterior talofibular ligament

Except for the extensor tendons there were both inconspicuous and pathological findings in all rated anatomical structures. The pathological findings in the study examinations, which are based on a consensus reading of the two readers on the well-established standard TSE imaging are listed in Table 2 (an exemplary lineup of structural pathologies is shown in Fig. 3).

**Table 2:** Present pathologies in the study collective [*n* = 47; *n* (1.5 T) = 22; *n* (3 T) = 25] defined by a consensus reading of two readers

	Item	1.5 T	3 T
Ligamentous structures 0-1-2-3 0 = intact 1 = posttraumatic alteration 2 = partial rupture 3 = complete rupture	aSyn	21-0-1-0	24-1-0-0
	pSyn	21-0-1-0	25-0-0-0
	ATFL	13-2-0-7	13-5-0-7
	CFL	15-3-0-4	15-5-0-5
	PTFL	18-3-1-0	23-0-1-1
	Delta	16-0-6-0	17-3-4-1
	Subtalar	20-0-1-1	22-1-1-1
	Chopart	19-2-0-1	21-1-3-0
Tendinous structures 0-1-2-3 0 = intact 1 = reactive alteration 2 = partial rupture 3 = complete rupture	Peroneal	22-0-0-0	21-2-2-0
	Flexor	20-2-0-0	22-3-0-0
	Extensor	22-0-0-0	25-0-0-0
	Achilles	20-1-0-1	23-1-0-1
	Plantar	21-1-0-0	24-1-0-0
Cartilage defects ICRS classification 0°–IVb°	Tibia	0°:22	0°:21 Ia°:1 II°:1 IVb:2
	Talus	0°:19 IIIa°:2 IVa°:1	0°:18 IIIa°:1 IIIc°:2 IVb°:4
OCL Kramer classification 0–5b	Talus	0:20 3a:1 3c:1	0:22 3a:1 4a:1 4b:1
Others Present–not present	BME Tibia	17-5	17-8
	BME Talus	16-6	15-10
	BME other	17-5	17-8
	Joint effusion	11-11	16-9
	STE	6-16	6-19

T Tesla, *aSyn* anterior syndesmosis, *pSyn* posterior syndesmosis, *ATFL* anterior talofibular ligament, *CFL* calcaneofibular ligament, *PTFL* posterior talofibular ligament, *Delta* delta ligament, *Subtalar* subtalar ligaments, *Chopart* chopart capsule, *Plantar* plantar fascia, *ICRS* International Cartilage Regeneration and Joint Preservation Society, *OCL* osteochondral lesions, *BME* bone marrow edema, *STE* soft tissue edema

### Image quality of standard TSE and DL TSE ankle MRI

**Fig. 4:**
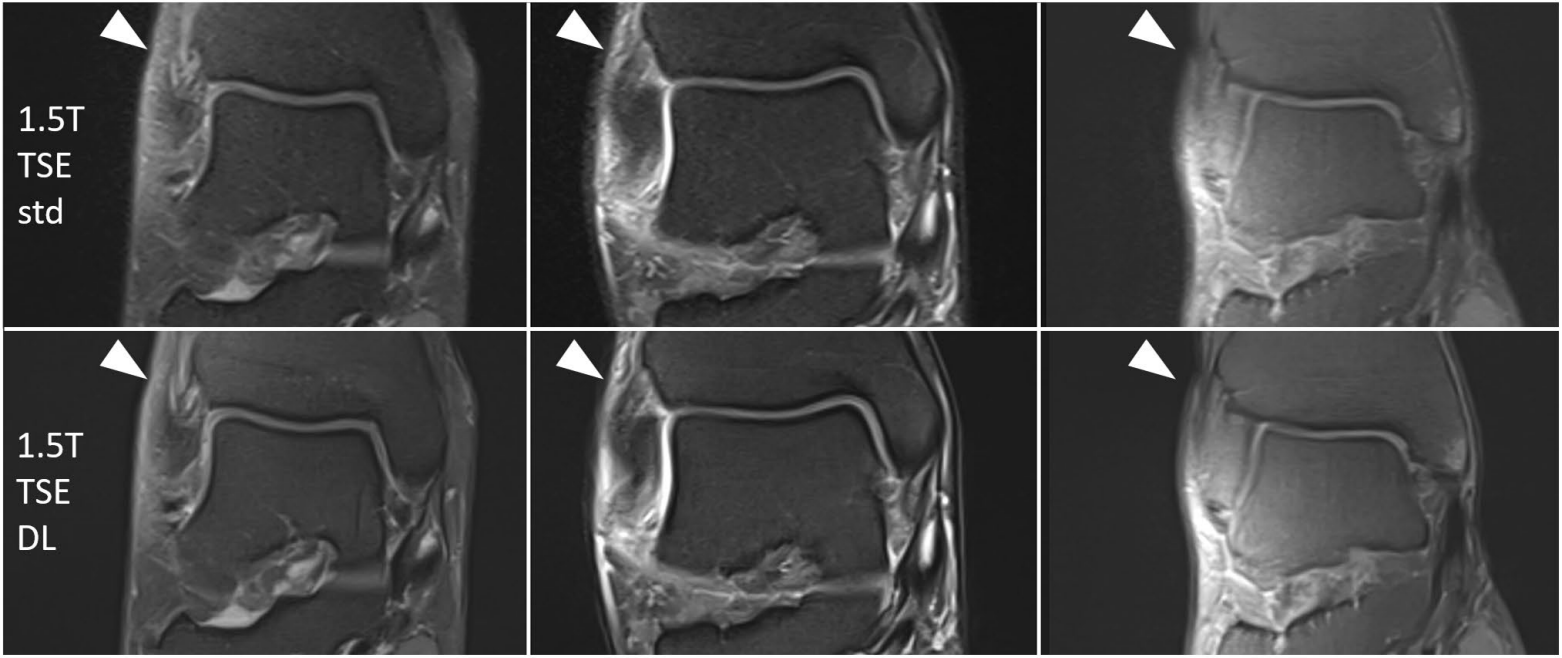
Exemplary 1.5 T MRI with reduced image noise and increased image sharpness in the new DL-based 2D TSE protocol (lower row) compared to the standard 2D TSE protocol (upper row; from left to right: right ankle, 56 years, female; right ankle, 28 years, male; right ankle, 27 years, male). T = Tesla, TSE = turbo spin echo, DL = deep learning, std = standard

**Fig. 5:**
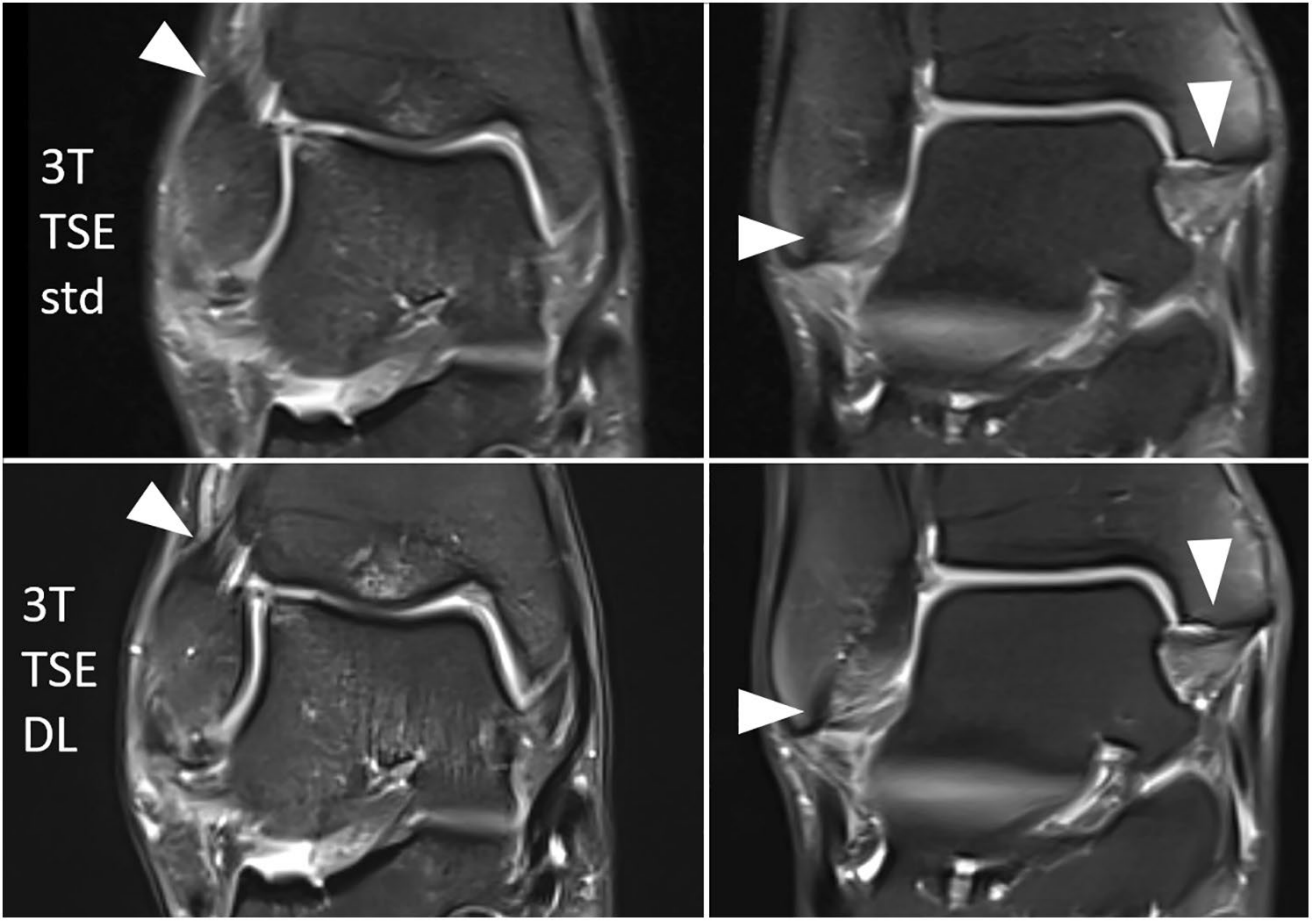
Exemplary 3 T MRI with slight differences regarding image noise and sharpness in the new DL-based 2D TSE protocol (lower row) compared to the standard 2D TSE protocol (upper row; from left to right: right ankle, 26 years, female; right ankle, 25 years, female). T = Tesla, TSE = turbo spin echo, DL = deep learning, std = standard

**Fig. 6:**
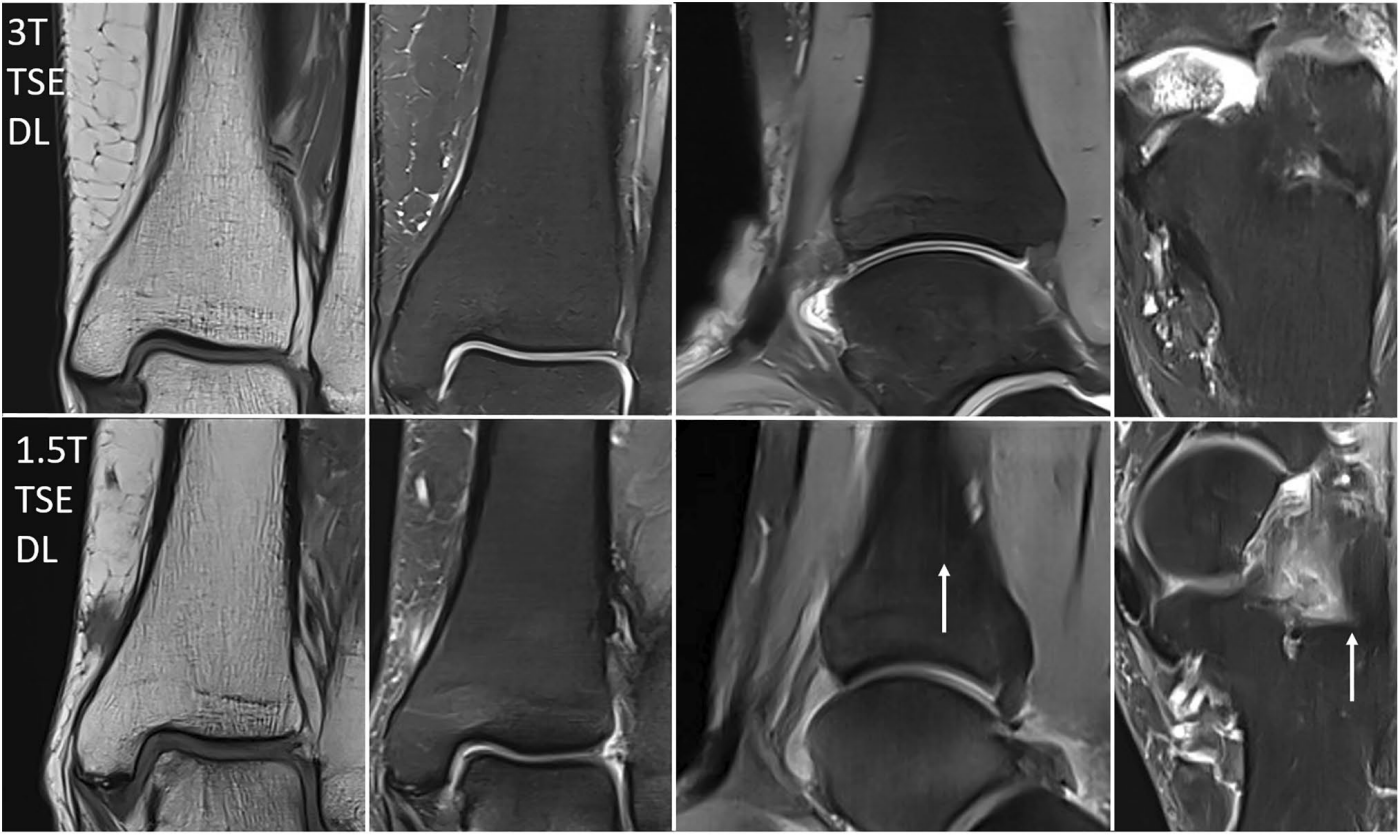
More distinct banding artifacts in 1.5 T DL TSE ankle MRI (lower row; left ankle, 57 years, female; from left to right: T1 coronal, PD with fat saturation coronal, sagittal, transversal) compared to 3 T DL TSE ankle MRI (upper row; left ankle, 19 years, female; from left to right: T1 coronal, PD with fat saturation coronal, sagittal, transversal). T = Tesla, TSE = turbo spin echo, DL = deep learning

In PD-weighted images, the DL TSE sequence showed significantly less image noise (1.5 T and 3 T), while banding artifacts were rated significantly worse (1.5 T and 3 T) and sharpness was rated slightly better for DL TSE without statistical significance (see Table 3 and exemplary patients’ images in Figs. 4, 5, 6).

**Table 3:** Image quality criteria of standard TSE and DL TSE on 1.5 T- and 3 T-ankle MRI (*n* = 47) independently rated by two radiologists

Sequence	Item	Reader	1.5 T			3 T		
			std	DL	*p*	std	DL	*p*
PD TSE	Noise	1	4 [2–5]	5 [3–5]	** < 0.0001**	5 [3–5]	5	0.05
		2	4 [2–5]	5 [3–5]	** < 0.0001**	5 [3–5]	5	**0.0237**
	Sharpness	1	4 [2–5]	4.5 [4–5]	0.16	5 [4–5]	5 [4–5]	0.33
		2	4 [2–5]	5 [4–5]	0.07	5 [4–5]	5 [4–5]	0.20
	Banding	1	5	4 [3–5]	** < 0.0001**	5	4 [3–5]	** < 0.0001**
		2	5 [4–5]	4 [3–5]	**0.0005**	5 [4–5]	4 [3–5]	** < 0.0001**
	DC	1	5 [3–5]	5 [4–5]	0.44	5	5	–
		2	5 [3–5]	5 [4–5]	0.31	5	5	–
T1 TSE	Noise	1	5	5	–	5	5	–
		2	5	5	–	5	5	–
	Sharpness	1	5 [4–5]	5 [4–5]	1.00	5	5 [4, 5]	0.23
		2	5 [4–5]	5 [4–5]	1.00	5	5 [3–5]	0.23
	Banding	1	5	5	–	5	3 [3–5]	** < 0.0001**
		2	5	5	–	5	4 [3–5]	** < 0.0001**
	DC	1	5	5	–	5	5	–
		2	5	5	–	5	5	–

Bold represents correlations were analyzed by likelihood ratio

*TSE* turbo spin echo, *DL* deep learning, *PD* proton density-weighted, *DC* diagnostic confidence, *std* standard

In the T1-weighted images banding artifacts were also rated worse for DL TSE on the 3 T scanners, but not on 1.5 T scanners. Other rated image quality criteria were not significantly different in T1-weighted images neither on 1.5 T nor on 3 T scanners.

In almost all patients, diagnostic confidence was rated excellent or good without any significant difference between standard TSE and DL TSE on 1.5 T scanners and 3 T scanners, respectively, as well as for PD-weighted or T1-weighted sequences (Table 3).

### Diagnostic accuracy of standard TSE and DL TSE ankle MRI

The intermethod agreement on all rated anatomical structures was almost perfect/perfect for both readers, both grouped regarding field strength (1.5 T and 3 T) and in total of the whole study collective. Thereby all anatomical structures in the group “Others” showed perfect agreement. For more details see Table 4.

**Table 4:** Intermethod agreement of standard TSE and DL TSE in 1.5 T and 3 T ankle MRI rated by two readers

	Item	Reader	κ 1.5 T	κ 3 T	κ overall
Ligamentous structures	aSyn	1	1.00	1.00	1.00
		2	1.00	1.00	1.00
	pSyn	1	1.00	x	1.00
		2	1.00	x	1.00
	AFTL	1	1.00	0.933	0.963
		2	0.953	0.926	0.939
	CFL	1	1.00	0.933	0.963
		2	0.908	0.926	0.918
	PFTL	1	1.00	1.00	1.00
		2	1.00	1.00	1.00
	Delta	1	1.00	1.00	1.00
		2	1.00	1.00	1.00
	Subtalar	1	1.00	1.00	1.00
		2	1.00	0.819	0.892
	Chopart	1	1.00	1.00	1.00
		2	1.00	1.00	1.00
Tendinous structures	Peroneal	1	x	1.00	1.00
		2	x	1.00	1.00
	Flexor	1	1.00	0.906	0.944
		2	1.00	0.815	0.889
	Extensor	1	x	x	x
		2	x	x	x
	Achilles	1	1.00	1.00	1.00
		2	1.00	1.00	1.00
	Plantar	1	1.00	1.00	1.00
		2	1.00	1.00	1.00
Cartilage defects	Tibia	1	x	1.00	1.00
		2	x	1.00	1.00
	Talus	1	1.00	0.911	0.942
		2	1.00	0.911	0.942
OCL	Talus	1	1.00	1.00	1.00
		2	1.00	1.00	1.00
Others	BME Tibia	1	1.00	1.00	1.00
		2	1.00	1.00	1.00
	BME Talus	1	1.00	1.00	1.00
		2	1.00	1.00	1.00
	BME other	1	1.00	1.00	1.00
		2	1.00	1.00	1.00
	Joint effusion	1	1.00	1.00	1.00
		2	1.00	1.00	1.00
	STE	1	1.00	1.00	1.00
		2	1.00	1.00	1.00

T Tesla, *aSyn* anterior syndesmosis, *pSyn* posterior syndesmosis, *ATFL* anterior talofibular ligament, *CFL* calcaneofibular ligament, *PTFL* posterior talofibular ligament, *Delta* delta ligament, *Subtalar* subtalar ligaments, *Chopart* chopart capsule, *Plantar* plantar fascia, *OCL* osteochondral lesions, *BME* bone marrow edema, *STE* soft tissue edema

## Discussion

The presented results show almost perfect/perfect agreement of DL TSE with standard TSE in all analyzed structure categories (ligamentous lesions, tendinous lesions, cartilage defects, OC, fluid sensitivity pathologies) with a median “good” or “excellent” rating in all image quality categories in both 1.5 T and 3 T MRI. The few discordant image interpretations referred to adjacent categories of the reading scheme and seemed to remain in the normal range of inter-individual image interpretation (illustrated for example in the image of the ATFL in the middle row of Fig. 3).

The reduction of total acquisition time of DL TSE compared to standard TSE was 49% in 1.5 T and 48% in 3 T MRI. There are no comparable studies on DL-based sequences in ankle MRI published yet, however, studies on the knee support our results of a high agreement of DL TSE with standard TSE maintaining realistic image impression, meanwhile saving a substantial amount of acquisition time [16, 17]. Though not in scope of the present study, the authors hypothesize that the results obtained here may be generalizable to other MSK imaging applications where similar contrasts are acquired i.e., hand-wrist or shoulder imaging.

The existence of banding artifacts in DL TSE sequences in MSK imaging has been reported in the literature [16]. While further algorithmic improvement of the DL reconstruction is desirable in this regard, the diminution of the image quality in the presented study was little.

Another interesting approach to accelerate ankle MRI imaging is the acquisition of 3D data sets with isotropic voxels [4–7]. The benefits are obvious: the acquisition of a single volume instead of coronal, sagittal and transversal data sets is supposed to save time, and additionally delivers the possibility for the reconstruction of oblique reformats for special issues. Previous works indicate that a high-quality data set with small voxel sizes (0.5 mm^3^) requires an acquisition time of about 10 min, what might be the reason why this approach has not been widely adopted in clinical practice. Moreover, simultaneous multi-slice excitation combined with PI has been reported an acceleration capability comparable to those of DL TSE combined with PI in the knee [23, 24]. Further studies have to prove the feasibility of multi-slice excitation techniques in the ankle. Besides the direct comparison of simultaneous multi-slice excitation sequences and conventional sequences, another comparison using DL-based reconstruction techniques would be interesting. Furthermore, DL-based reconstructions of simultaneous multi-slice excitation sequences are promising [25].

Limitations of the presented study were the single center design and different MRI scanners from a single vendor, however, it in turn shows that the results may not be restricted to specific MR scanners and receive coils of this vendor. Suitable studies could substantiate the auspicious results on DL TSE in ankle MRI.

In conclusion, DL TSE is a promising new technique for 1.5 T and 3 T ankle MRI with high agreement compared to standard TSE and excellent image impression while saving 48% and 49% acquisition time.

## Abbreviations

2D: Two-dimensional
3D: Three-dimensional
aSyn: Anterior syndesmosis
ATFL: Anterior talofibular ligament
Av: Averages
BME: Bone marrow edema
C: Concatenations
CFL: Calcaneofibular ligament
CS: Compressed sensing
DC: Diagnostic confidence
DL: Deep learning
FA: Flip angle
FOV: Field of view
ICRS: International Cartilage Regeneration and Joint Preservation Society
MRI: Magnetic resonance imaging
MSK: Musculoskeletal
OCL: Osteochondral lesion
PAT: Parallel acquisition technique factor
PD: Proton density
PI: Parallel imaging
pSyn: Posterior syndesmosis
PTFL: Posterior talofibular ligament
SNR: Signal-to noise ratio
std: Standard
STE: Soft tissue edema
T: Tesla
TA: Acquisition time
TE: Time to echo
TF: Turbo factor
TR: Time to repeat
TSE: Turbo spin echo
